# Risk Factors for Death Among Hospitalized Patients Aged 21–64 Years Diagnosed with COVID-19—New York City, March 13–April 9, 2020

**DOI:** 10.1007/s40615-021-01098-1

**Published:** 2021-08-09

**Authors:** Dena Bushman, Alexander Davidson, Preeti Pathela, Sharon K. Greene, Don Weiss, Vasudha Reddy, New York City Fatal Case-Control Study Team, Julia Latash

**Affiliations:** 1 New York City Department of Health and Mental Hygiene, Queens, NY USA; 2 Epidemic Intelligence Service, Centers for Disease Control and Prevention, Atlanta, GA USA

**Keywords:** COVID-19, SARS-CoV-2, New York City, Risk factors, Racial/ethnic disparities, In-hospital mortality

## Abstract

**Background:**

COVID-19 mortality studies have primarily focused on persons aged ≥ 65 years; less is known about decedents aged <65 years.

**Methods:**

We conducted a case-control study among NYC residents aged 21–64 years hospitalized with COVID-19 diagnosed March 13–April 9, 2020, to determine risk factors for death. Case-patients (n=343) were hospitalized decedents with COVID-19 and control-patients (n=686) were discharged from hospitalization with COVID-19 and matched 2:1 to case-patients on age and residential neighborhood. Conditional logistic regression models were adjusted for patient sex, insurance status, and marital status. Matched adjusted odds ratios (aORs) were calculated for selected underlying conditions, combinations of conditions, and race/ethnic group.

**Results:**

Median age of both case-patients and control-patients was 56 years (range: 23–64 years). Having ≥ 1 selected underlying condition increased odds of death 4.45-fold (95% CI: 2.33–8.49). Patients with diabetes; morbid obesity; heart, kidney, or lung disease; cancer; neurologic/neurodevelopmental conditions; mental health conditions; or HIV had significantly increased odds of death. Compared with having neither condition, having both diabetes and obesity or diabetes and heart disease was associated with approximately threefold odds of death. Five select underlying conditions were more prevalent among non-Hispanic Black control-patients than among control-patients of other races/ethnicities.

**Conclusions and Relevance:**

Selected underlying conditions were risk factors for death, and most prevalent among racial/ethnic minorities. Social services; health care resources, including vaccination; and tailored public health messaging are important for COVID-19 prevention. Strengthening these strategies for racial/ethnic minority groups could minimize COVID-19 racial/ethnic disparities.

## Introduction

New York City (NYC) was an early United States (U.S.) epicenter of the coronavirus disease 2019 (COVID-19) pandemic [1]. By April 30, 2020, 174,501 confirmed COVID-19 cases, 47,779 hospitalizations, and 14,905 deaths occurred among NYC residents [2]. During this time, NYC public health messaging encouraged the public to stay home [3–7], possibly contributing to delays in obtaining emergency care [8, 9].

Among persons hospitalized with COVID-19 in the U.S., underlying conditions, including chronic kidney disease, cardiovascular disease, immunosuppression, chronic lung disease, neurologic disorders, diabetes, and obesity, have been identified as risk factors for mortality [10–13]. NYC-based COVID-19 in-hospital mortality studies have primarily used data from single hospital systems or focused on older persons, for whom elevated mortality rates have been observed [12–19]. However, many deaths in NYC were reported among persons aged 21–64 years. By April 30, 2020, such persons accounted for nearly 26% of COVID-19–related mortality [20]. In the U.S., COVID-19 diagnoses and hospitalizations have disproportionately impacted communities of color [1, 11, 21–23]. While age-adjusted COVID-19 hospitalization and mortality rates in NYC have been higher among Hispanic/Latino (1060 and 298 per 100,000, respectively) and non-Hispanic Black (988 and 274 per 100,000, respectively) persons compared with non-Hispanic White (531 and 155 per 100,000, respectively) and non-Hispanic Asian (493 and 140 per 100,000, respectively) persons, higher in-hospital mortality risk among persons of color in NYC has not been previously reported [12, 13, 18, 19, 24].

We examined underlying conditions, stratified by race/ethnicity and age, to assess risk factors associated with death among NYC residents aged 21–64 years hospitalized with COVID-19. These findings can inform clinicians and public health professionals in allocating resources and tailoring preventive measures during the COVID-19 pandemic.

## Methods

### Data Sources

Laboratories are required to electronically report SARS-CoV-2 test results for NYC residents to the NY State Electronic Clinical Laboratory Reporting System [25]. To obtain hospitalization status, the NYC Department of Health and Mental Hygiene (DOHMH) matched patient identifiers from laboratory reports of confirmed COVID-19 cases with emergency department syndromic surveillance, the NY State Hospital Emergency Response Data System, regional health information organizations, NYC public hospitals, and DOHMH’s death registry [1]. On March 26, 2020, the DOHMH requested remote access to electronic health records (EHRs) for patients hospitalized with COVID-19 in selected NY metropolitan area hospitals. Eighty hospitals and two temporary hospitals agreed, representing facilities where 89% of decedents between February 22 and April 9, 2020, were first hospitalized with COVID-19.

Investigators used a medical chart abstraction guidance document and an investigation form to abstract patients’ demographics, substance use, occupation, past medical history, and clinical course. Substance use and occupation were inconsistently documented in EHRs and omitted from analyses. Presence of underlying conditions/symptoms was collected as “Indicated” when documented in EHRs, otherwise “Not indicated,” because absence of documentation could not be assumed to mean absence of those conditions/symptoms. Categories of underlying conditions were created for (1) conditions prevalent among persons with COVID-19 [17, 21, 26] and (2) conditions commonly abstracted by investigations that did not fit existing categories. Data quality was supported by two independent investigators performing each abstraction. Study patients were matched [27] with the tuberculosis, HIV/AIDS, and hemoglobin A1c DOHMH registries [28] using standard key deterministic algorithms, and with the hepatitis B and C registries using a patient-level unique identifier. This activity was reviewed by the CDC and was conducted consistent with applicable federal law and CDC policy [1]. The DOHMH Institutional Review Board determined this activity to be non-research public health surveillance.

### Analytic Sample

We conducted a matched case-control study of NYC residents aged 21–64 years who were reported to the DOHMH with nucleic acid amplification test–confirmed COVID-19 [29] during March 13–April 9, 2020, and were hospitalized ≥1 time for reasons other than labor and delivery in facilities for which the DOHMH had EHR access. Sample size calculations for tests for two correlated proportions in a matched case-control design [30] were conducted using PASS 2019 (v19.0.3, NCSS, LLC, Kaysville, UT) (Appendix 1).

Among 15,097 eligible persons who met case-patient eligibility criteria described above, 1698 (11.2%) died in an emergency department (ED) or hospital by April 13. Of these, 45 (2.7%) were excluded because they died at an unknown location or outside of the hospital. Of the remaining 1653 eligible decedents, 350 were randomly selected, and medical chart abstraction was completed for 343 case-patients.

Control-patients met laboratory and hospitalization eligibility criteria described above, and were discharged alive from hospitalization(s). Matching was performed to reduce variance of effect estimates [31]. Control-patients were matched 2:1 to case-patients on age (± 3 years, within the bounds of 21–64) and neighborhood of residence, as defined by the NYC United Hospital Fund [32]. Control-patients who left hospitals against medical advice or who were discharged to nursing homes, group homes, or step-down facilities were considered “discharged” and were included if they met the other inclusion criteria. Those discharged to hospice or still receiving inpatient hospital care at the time of medical chart abstraction were excluded and substituted with other matched control-patients. Among 13,399 eligible persons, medical chart abstractions were completed for 686 randomly selected, matched control-patients. Through a citywide death registry match, no known deaths among control-patients were documented as of July 1, 2020, when data collection was completed.

### Data Analysis

To assess generalizability, given lack of EHR access to all NY metropolitan hospitals, we calculated relative risks (RRs) and 95% confidence intervals (CIs) to compare whether key demographics of case-patients differed significantly from other decedents who died in hospitals or EDs (including hospitals for which the DOHMH did not have EHR access) not selected for the study.

All case-patients with identical matching criteria and their matched controls were pooled, totaling 260 strata. We conducted conditional logistic regression analyses to evaluate associations between selected underlying conditions and odds of death and presented matched adjusted odds ratios (aORs) and 95% CIs. All models included one condition of a priori interest and adjusted for sex (male, female) and two covariates thought to be potential confounders and significant in bivariate analyses: insurance status (private insurance, public insurance, uninsured) and marital status (married, not married).

Patient residence was assigned a neighborhood poverty level, defined as the percent of the population living in a given census tract whose household income was below the federal poverty level (FPL) per American Community Survey 2014–2018, with low poverty being <10.0% below FPL, medium poverty 10.0–19.9% below FPL, high poverty 20.0–29.9% below FPL, and very high poverty ≥30.0% below FPL [33]. Obesity, a key exposure of interest, was considered in multiple ways. Body mass index (BMI) was calculated when height and weight were known. A five-level categorical variable for obesity was created based on National Institutes of Health (NIH) criteria: “Underweight” (<18.5 kg/m^2^), “Normal weight” (18.5–<25.0 kg/m^2^), “Overweight” (25.0–<30.0 kg/m^2^), “Obese” (30.0–<40.0 kg/m^2^), and “Morbidly obese” (≥40.0 kg/m^2^) [34]. A two-level categorical obesity variable (“Indicated” vs. “Not indicated”) was also created; obesity was “Indicated” if BMI was ≥ 30 kg/m^2^ or BMI was unknown, but obesity was documented in the study patient’s chart.

We described clinical characteristics of study patients, including presentation of symptoms, diagnoses of pneumonia and acute respiratory distress syndrome, use of respiratory support, initiation of dialysis, admission to the intensive care unit, and use of inpatient treatments. We evaluated combinations of comorbidities as risk factors for death by assessing the odds of death among patients with two specified underlying conditions. Conditions chosen for these analyses were prevalent (occurring among approximately ≥10% of case-patients) and significantly associated with death when adjusting for sex, marital status, and insurance status, and for which, directionality of causation is not always clear [35–39]. We analyzed combinations of conditions as four-level categorical variables, with patients having neither, one, or both conditions.

To assess racial/ethnic disparities in associations between selected underlying conditions and odds of death, we stratified analyses by the following racial/ethnic groups: non-Hispanic Asian (Asian), non-Hispanic Black (Black), Hispanic/Latino (Hispanic), non-Hispanic White (White), and non-Hispanic (Other), which included non-Hispanic patients of other or multiple races. Patients of other races were included in regression models, but effect estimates for this group are not presented because of sparsity. We present one aOR for each racial/ethnic group per condition, comparing patients with the condition against patients for whom the condition was not indicated. To determine which conditions to include, we used the following criteria: any significant overall aOR with approximately ≥10% of case-patients having the condition, or, if not statistically significant, an overall aOR ≥1.5 and ≥15% of case-patients having the condition. Because sparse data within some strata could contribute to underpowered analyses, we do not present stratified aORs for specific conditions that were part of grouped categories (e.g., hypertension as a subset of heart disease). To help explain racial/ethnic disparities in COVID-19 deaths in NYC, we assessed prevalence of underlying conditions by race/ethnicity among control-patients. Given minimal data collected during the pandemic on underlying condition prevalence among non-hospitalized COVID-19 patients, control-patients were the best available proxy for NYC’s population to understand risk factors for in-hospital mortality with COVID-19. Similarly, to assess differences in associations between selected conditions and odds of death among those aged <50 years and ≥50 years, we calculated aORs restricted to each age group.

All analyses were conducted using SAS version 9.4 (Cary, NC).

## Results

Distributions of age, sex, borough of residence, and neighborhood-level poverty for case-patients (n = 343) were similar to other decedents who died in hospitals or EDs (including hospitals for which the DOHMH did not have EHR access) but were not selected for the study (n = 1310). Asian race/ethnicity was the only demographic characteristic that was significantly differentially distributed between case-patients (9.1%) and decedents not selected for the study (5.0%) (RR, 1.52 [95% CI: 1.04, 2.23]).

Among study patients, the median age of both case-patients and control-patients was 56 years (range: 23–64 years) (Table 1); 95.2% of control-patients matched case-patients on age exactly (± 0 years). Overall, patients were predominantly male (65.5%), Hispanic (37.6%) or Black (29.1%), and residents of Queens (37.1%) or the Bronx (29.8%), and resided in medium poverty neighborhoods (37.0%); these characteristics were similarly distributed among case-patients and control-patients, as expected from matching on residential neighborhood. However, case-patients were more likely to be unmarried (54.9% vs. 44.6%) and less likely to have private health insurance (26.8% vs. 41.8%).

**Table 1:** Matched odds ratios (mOR) comparing demographic information of study patients (N = 1029), by patient status—New York City, March 13–April 9, 2020

Characteristic	Cases (n = 343)	Controls (n = 686)	Total (n = 1029)	mOR (95% CI)^1^
Median age (range)^2^	56 (23–64)	56 (23–64)	56 (23–64)	
Sex—n (%)				
Female	113 (32.9)	242 (35.3)	355 (34.5)	0.90 (0.68, 1.19)
Male	230 (67.1)	444 (64.7)	674 (65.5)	Reference
Race/ethnicity—n (%)				
Non-Hispanic Asian	31 (9.1)	60 (8.9)	91 (9.5)	0.92 (0.49, 1.72)
Non-Hispanic Black	109 (31.9)	187 (27.7)	296 (29.1)	1.13 (0.69, 1.86)
Hispanic/Latino	120 (35.1)	262 (38.9)	382 (37.6)	0.81 (0.49, 1.33)
Non-Hispanic White	48 (14.0)	98 (14.5)	146 (14.4)	Reference
Non-Hispanic Other^3^	34 (9.9)	67 (9.9)	101 (12.9)	
Unknown^4^	1	12	13	
Borough of residence^2^—n (%)				
Bronx	103 (30.0)	204 (29.7)	307 (29.8)	
Brooklyn	76 (22.2)	150 (21.9)	226 (22.0)	
Manhattan	21 (6.1)	45 (6.6)	66 (6.4)	
Queens	127 (37.0)	255 (37.2)	382 (37.1)	
Staten Island	16 (4.7)	32 (4.7)	48 (4.7)	
Proportion of population in census tract of residence living below the federal poverty level^4^—n (%)				
≥30.0% (very high poverty)	82 (24.1)	145 (21.3)	227 (22.2)	1.58 (0.93, 2.68)
20.0% – <30.0% (high poverty)	72 (21.1)	145 (21.3)	217 (21.3)	1.27 (0.80, 2.01)
10.0% – <20.0% (medium poverty)	127 (37.2)	251 (36.9)	378 (37.0)	1.22 (0.82, 1.81)
<10.0% (low poverty)	60 (17.6)	141 (20.7)	201 (19.7)	Reference
Unknown^4^	2	6	8	
Marital status—n (%)				
Not married	178 (54.9)	296 (44.6)	474 (48.0)	**1.53 (1.16, 2.02)**
Married	146 (45.1)	368 (55.4)	514 (52.0)	Reference
Unknown^4^	19	22	41	
Insurance status—n (%)				
Public	202 (58.9)	308 (44.9)	510 (49.6)	1.22 (0.82, 1.83)
Private	92 (26.8)	287 (41.8)	379 (36.8)	**0.58 (0.38, 0.90)**
Uninsured	49 (14.3)	91 (13.3)	140 (13.6)	Reference
Pregnant^6^				
Indicated	0 (0.0)	3 (1.2)	3 (0.8)	**--**^**7**^
Not indicated	113 (100.0)	239 (98.8)	352 (99.2)	

^1^Bold-faced mORs indicate significance at alpha=0.05

^2^Odds ratios not produced for age or borough of residence, as study population was matched on age and United Hospital Fund neighborhood

^3^Non-Hispanic Other is included in the table and included in the regression model, but no OR is shown because the effect estimate has no meaningful interpretation

^4^Unknown numbers are shown in the table but were not included in the regression model

^5^American Community Survey 2014–2018 census tract data (defined according to 2010 boundaries)

^6^Restricted to female patients

^7^Undefined effect estimate

### Hospitalization Course

Most patients were known to be symptomatic prior to or during hospitalization (99.8%); the most common symptoms were fever (90.5%), shortness of breath (87.9%), and cough (86.5%) (Table 2). The median numbers of days from symptom onset to diagnosis and from symptom onset to first hospitalization were both 5 days for case-patients and 7 days for control-patients. Among case-patients, 95.3% and 58.9% had a diagnosis of pneumonia and acute respiratory distress syndrome, respectively, compared with 91.0% and 9.6% of control-patients. Most case-patients (86.3%) and control-patients (83.8%) received supplemental oxygen, and 78.7% of case-patients and 9.8% of control-patients were intubated and placed on a mechanical ventilator. Among case-patients (90.1%) and control-patients (96.5%) who did not report being on dialysis at time of hospitalization, dialysis was initiated for 13.1% and 2.5% of case-patients and control-patients respectively. ICU admission occurred for 66.7% of case-patients and 14.9% of control-patients. The most common inpatient treatments administered to case-patients and control-patients were hydroxychloroquine (88.1% and 81.5%, respectively), azithromycin (85.7% and 72.2%), corticosteroids (30.0% and 18.8%), and ascorbic acid (21.9% and 23.0%).

**Table 2:** Clinical information for study patients (N = 1029), by patient status—New York City, March 13–April 9, 2020

Clinical variables	Cases (n=343)	Controls (n=686)	Total (n=1029)
	Number (%)	Number (%)	Number (%)
Symptomatic^1^	342 (99.7)	685 (99.9)	1027 (99.8)
Median number of days from symptom onset to diagnosis in days (range)^2^	5 (0–45)	7 (0–31)	6 (0–45)
Symptoms			
Fever	300 (87.7)	629 (91.8)	929 (90.5)
Shortness of breath	316 (92.4)	587 (85.7)	903 (87.9)
Cough	273 (79.8)	615 (89.9)	888 (86.5)
Fatigue	132 (38.6)	351 (51.2)	483 (47.0)
Myalgia	121 (35.4)	351 (51.2)	472 (46.0)
Chills	106 (31.0)	319 (46.6)	425 (41.4)
Diarrhea	79 (23.1)	253 (36.9)	332 (32.3)
Nausea/vomiting	73 (21.4)	220 (32.1)	293 (28.5)
Chest pain	53 (15.5)	199 (29.1)	252 (24.5)
Anorexia	46 (13.4)	189 (27.6)	235 (22.9)
Headache	46 (13.4)	137 (20.0)	183 (17.8)
Sore throat	36 (10.5)	122 (17.8)	158 (15.4)
Abdominal pain	36 (10.5)	120 (17.5)	156 (15.2)
Altered mental status	86 (25.2)	62 (9.1)	148 (14.4)
Nasal congestion	30 (8.8)	100 (14.6)	130 (12.7)
Dizziness	27 (7.9)	85 (12.4)	112 (10.9)
Sweating	20 (5.9)	34 (5.0)	54 (5.3)
Loss of taste	4 (1.2)	26 (3.8)	30 (2.9)
Loss of smell	3 (0.9)	26 (3.8)	29 (2.8)
Bloody sputum	9 (2.6)	17 (2.5)	26 (2.5)
Rigors	4 (1.2)	12 (1.8)	16 (1.6)
Hospitalization course^3^			
Time to hospitalization^4^			
Median number of days from symptom onset to first hospitalization admission (range)	5 (0–32)	7 (0–43)	6 (0–43)
Pneumonia	327 (95.3)	624 (91.0)	951 (92.4)
Acute respiratory distress syndrome	202 (58.9)	66 (9.6)	268 (26.0)
Respiratory support			
Supplemental oxygen	296 (86.3)	575 (83.8)	871 (84.6)
Intubation/mechanical ventilation	270 (78.7)	67 (9.8)	337 (32.8)
Continuous positive airway pressure/bilevel positive airway pressure	68 (19.8)	38 (5.5)	103 (10.0)
Extracorporeal membrane oxygenation	1 (0.3)	1 (0.2)	2 (0.2)
Dialysis initiated	45 (13.1)	17 (2.5)	62 (6.0)
Intensive care unit admission	229 (66.7)	102 (14.9)	331 (32.2)
Median length of stay in days (range)	4 (0–26)	8.5 (0–55)	5 (0–55)
Inpatient treatment			
Hydroxychloroquine	302 (88.1)	559 (81.5)	861 (83.7)
Azithromycin	294 (85.7)	495 (72.2)	789 (76.7)
Ascorbic acid	75 (21.9)	158 (23.0)	233 (22.6)
Corticosteroids	103 (30.0)	129 (18.8)	232 (22.5)
Tocilizumab^5^	26 (7.6)	27 (3.9)	53 (5.2)
Oseltamivir	24 (7.0)	35 (5.1)	59 (5.7)
Angiotensin-converting enzyme inhibitor	9 (2.6)	15 (2.2)	24 (2.3)
Lopinavir/ritonavir	7 (2.0)	10 (1.4)	17 (1.7)
Angiotensin II receptor blockers	6 (1.8)	12 (1.8)	18 (1.7)
Remdesivir^5^	5 (1.5)	9 (1.3)	14 (1.4)
Sarilumab^5^	4 (1.2)	9 (1.3)	13 (1.3)
Chloroquine	3 (0.9)	6 (0.9)	9 (8.7)
Convalescent plasma^5^	3 (0.9)	7 (1.0)	10 (9.7)

^1^A patient was considered symptomatic if symptoms were noted in the EHR

^2^Based on 325 case-patients and 675 control-patients for whom onset date was available

^3^Some case-patients and control-patients were hospitalized more than once with COVID-19 within the study period. Hospitalization course data includes all hospitalizations with COVID-19 that occurred within the study period

^4^Time to hospitalization calculated as the number of days between symptom onset date and first hospitalization admission. This calculation is restricted to case-patients (n=320) and control-patients (n=676) with symptoms noted in the EHR, for whom symptom onset date was known, and for whom symptoms began before initial hospitalization

^5^Includes patients participating in clinical trials of this drug and who might have received placebo

### Underlying Conditions

Most case-patients (96.5%) and control-patients (86.4%) had ≥1 underlying condition (Table 3). After adjusting for sex, marital status, and insurance status, having ≥1 selected underlying condition was strongly associated with death (aOR, 4.45 [95% CI: 2.33, 8.49]). Increased odds of death were observed for patients with diabetes (aOR, 2.09 [95% CI: 1.56, 2.78]) and obesity (aOR,1.62 [95% CI: 1.22, 2.14]), particularly morbid obesity (versus patients with normal BMI (aOR, 3.07 [95% CI: 1.59, 5.90]). Heart disease was associated with an increased odds of death (aOR, 2.16 [95% CI: 1.55, 3.01]); specifically, history of heart attack, congestive heart failure, coronary artery disease, and hypertension were each significantly associated with increased odds of death. Lung disease was associated with an increased odds of death (aOR, 1.52 [95% CI: 1.08, 2.14]). Specifically, having chronic obstructive pulmonary disease/chronic bronchitis/emphysema was significantly associated with an increased odds of death (aOR, 2.40 [95% CI: 1.39, 4.16]). Asthma was not significantly associated with death (aOR, 1.22 [95% CI: 0.82, 1.81]). Patients with kidney disease had 2.51 (95% CI: 1.72, 3.68) times the odds of death compared with those without kidney disease. Other underlying conditions significantly associated with death included cancer, neurologic or neurodevelopmental condition(s), mental health condition(s), anemia, and HIV/AIDS.

**Table 3:** Matched adjusted odds ratios (aOR) of death with COVID-19 among study patients (N = 1029) with specific underlying medical conditions—New York City, March 13–April 9, 2020

Condition^1^	Cases (n = 343)	Controls (n = 686)	Matched aOR (95% CI)^2^
Any underlying condition—n (%)			
Indicated	331 (96.5)	593 (86.4)	**4.45 (2.33, 8.49)**
Not indicated	12 (3.5)	93 (13.6)	Reference
Heart disease^3^—n (%)			
Indicated	259 (75.5)	416 (60.6)	**2.16 (1.55, 3.01)**
Not indicated	84 (24.5)	270 (39.4)	Reference
Hypertension—n (%)			
Indicated	232 (67.6)	351 (51.2)	**2.08 (1.53, 2.82)**
Not indicated	111 (32.4)	335 (48.8)	Reference
Hyperlipidemia—n (%)			
Indicated	130 (37.9)	240 (35.0)	1.10 (0.81, 1.50)
Not indicated	213 (62.1)	446 (65.0)	Reference
Coronary artery disease—n (%)			
Indicated	58 (16.9)	64 (9.3)	**1.66 (1.10, 2.51)**
Not indicated	285 (83.1)	622 (90.7)	Reference
History of heart attack—n (%)			
Indicated	35 (10.2)	21 (3.1)	**3.41 (1.87, 6.23)**
Not indicated	308 (89.8)	665 (96.9)	Reference
Congestive heart failure—n (%)			
Indicated	33 (9.6)	34 (5.0)	**1.84 (1.05, 3.22)**
Not indicated	310 (90.4)	652 (95.0)	Reference
Valvular heart disease—n (%)			
Indicated	19 (5.5)	16 (2.3)	1.85 (0.88, 3.87)
Not indicated	324 (94.5)	670 (97.7)	Reference
Congenital heart disease—n (%)			
Indicated	3 (0.9)	4 (0.6)	1.61 (0.32, 8.06)
Not indicated	340 (99.1)	682 (99.4)	Reference
Obesity—n (%)			
Indicated	209 (60.9)	346 (50.4)	**1.62 (1.22, 2.14)**
Not indicated	134 (39.1)	340 (49.6)	Reference
Body mass index category^4^—n (%)			
Underweight (<18.5 kg/m^2^)	3 (1.2)	3 (0.6)	1.80 (0.34, 9.66)
Normal weight (18.5– <25.0 kg/m^2^)	38 (14.8)	84 (15.4)	Reference
Overweight (25.0 – <30.0 kg/m^2^)	69 (26.8)	195 (35.7)	0.88 (0.52, 1.50)
Obese (30.0– <40.0 kg/m^2^)	98 (38.1)	217 (39.7)	1.19 (0.72, 1.96)
Morbidly obese (≥40.0 kg/m^2^)	49 (19.1)	47 (8.6)	**3.07 (1.59, 5.90)**
BMI unknown	86	140	
Diabetes—n (%)			
Indicated	192 (56.0)	250 (36.4)	**2.09 (1.56, 2.78)**
Type 1	2 (0.6)	3 (0.4)	
Type 2	153 (43.7)	226 (32.9)	
Unknown	37 (10.8)	21 (3.1)	
Not indicated	151 (44.0)	250 (63.6)	Reference
Lung disease^5^—n (%)			
Indicated	90 (26.2)	131 (19.1)	**1.52 (1.08, 2.14)**
Not indicated	253 (73.8)	555 (80.9)	Reference
Asthma—n (%)			
Indicated	57 (16.62)	95 (13.8)	1.22 (0.82, 1.81)
Not indicated	286 (83.38)	591 (86.2)	Reference
Chronic obstructive pulmonary disease/bronchitis/emphysema—n (%)			
Indicated	34 (9.9)	29 (4.2)	**2.40 (1.39, 4.16)**
Not indicated	309 (90.1)	657 (95.8)	Reference
Cystic fibrosis—n (%)			
Indicated	0	0	--^6^
Not indicated	343 (100.0)	686 (100.0)	
Kidney disease^7^—n (%)			
Indicated	79 (23.0)	71 (10.3)	**2.51 (1.72, 3.68)**
Not indicated	264 (77.0)	615 (89.7)	Reference
Renal failure on dialysis—n (%)			
Indicated	34 (9.9)	24 (3.5)	**2.73 (1.53, 4.88)**
Not indicated	309 (90.1)	662 (96.5)	Reference
Neurologic/neurodevelopmental condition^8^—n (%)			
Indicated	65 (19.0)	56 (8.2)	**2.42 (1.58, 3.69)**
Not indicated	278 (81.0)	630 (91.8)	Reference
Mental health condition^9^—n (%)			
Indicated	43 (12.5)	53 (7.7)	**1.61 (1.02, 2.54)**
Not indicated	300 (87.5)	633 (92.3)	Reference
Cancer—n (%)			
Indicated	37 (10.8)	32 (4.7)	**2.54 (1.52, 4.23)**
Diagnosed <1 year ago	11 (3.2)	7 (1.0)	
Diagnosed 1 or more years ago	26 (7.6)	25 (3.6)	
Not indicated	306 (89.2)	654 (95.3)	Reference
Thyroid disorder—n (%)			
Indicated	33 (9.6)	47 (6.9)	1.38 (0.84, 2.27)
Not indicated	310 (90.4)	639 (93.1)	Reference
Anemia—n (%)			
Indicated	32 (9.3)	41 (6.0)	**1.69 (1.01, 2.80)**
Not indicated	311 (90.7)	645 (94.0)	Reference
Obstructive sleep apnea—n (%)			
Indicated	32 (9.3)	43 (6.3)	1.49 (0.91, 2.46)
Not indicated	311 (90.7)	643 (93.7)	Reference
Peripheral vascular disease—n (%)			
Indicated	29 (8.5)	32 (4.7)	1.59 (0.89, 2.84)
Not indicated	314 (91.5)	654 (95.3)	Reference
Liver disease^10^—n (%)			
Indicated	24 (7.0)	49 (7.1)	0.75 (0.44, 1.27)
Not indicated	319 (93.0)	637 (92.9)	Reference
Viral hepatitis—n (%)			
Indicated	17 (5.0)	28 (4.1)	0.87 (0.45, 1.67)
Not indicated	326 (95.0)	658 (95.9)	Reference
Hepatitis C virus infection—n (%)			
Indicated	10 (2.9)	17 (2.5)	0.82 (0.36, 1.88)
Not indicated	333 (97.1)	669 (97.5)	Reference
Hepatitis B virus infection—n (%)			
Indicated	9 (2.6)	11 (1.6)	1.28 (0.48, 3.40)
Not indicated	334 (97.4)	675 (98.4)	Reference
Cirrhosis			
Indicated	7 (2.0)	13 (1.9)	0.85 (0.33, 2.21)
Not indicated	336 (98.0)	673 (98.1)	Reference
Nonalcoholic fatty liver disease—n (%)			
Indicated	2 (0.6)	9 (1.3)	0.44 (0.09, 2.07)
Not indicated	341 (99.4)	677 (98.7)	Reference
History of stroke—n (%)			
Indicated	23 (6.7)	27 (3.9)	1.43 (0.78, 2.62)
Not indicated	320 (93.3)	659 (96.1)	Reference
Autoimmune disease—n (%)			
Indicated	22 (6.4)	29 (4.2)	1.70 (0.93, 3.10)
Not indicated	321 (93.6)	657 (95.8)	Reference
Gastroesophageal reflux disorder—n (%)			
Indicated	19 (5.5)	57 (8.3)	0.65 (0.36, 1.16)
Not indicated	324 (94.5)	629 (91.7)	Reference
HIV/AIDS			
Indicated	15 (4.4)	13 (1.9)	**2.42 (1.05, 5.59)**
AIDS	3 (0.9)	3 (0.4)	
AIDS not indicated	12 (3.5)	10 (1.5)	
Not indicated	328 (95.6)	673 (98.1)	Reference
History of organ transplant—n (%)			
Indicated	8 (2.3)	6 (0.9)	2.97 (0.95, 9.27)
Not indicated	335 (97.7)	680 (99.1)	Reference
Active tuberculosis—n (%)			
Indicated	1 (0.3)	0	--^6^
Not indicated	342 (99.7)	686 (100.0)	

^1^Conditions listed in descending order of prevalence among case-patients

^2^Adjusted for sex, marital status, and insurance status. Bold-faced aORs indicate significance at alpha=0.05

^3^Includes history of heart attack, congestive heart failure, coronary artery disease, hypertension, hyperlipidemia, congenital heart disease, valvular heart disease, and other chronic heart conditions (such as angina, arrhythmias, cardiomyopathy, and pulmonary hypertension)

^4^Defined according to National Institutes of Health criteria, weight (kg)/height (m^2^)

^5^Includes asthma, chronic obstructive pulmonary disease, chronic bronchitis, emphysema, cystic fibrosis, and other chronic lung conditions (such as pulmonary nodules, pulmonary fibrosis, and history of pulmonary emboli)

^6^Undefined effect estimate

^7^Includes chronic kidney conditions (such chronic kidney disease, glomerulonephritis, and polycystic kidney disease)

^8^Includes conditions such as seizure disorders, intellectual disability, dementia, history of traumatic brain injury, cerebral palsy, and neuropathy

^9^Includes conditions such as attention deficit hyperactivity disorder, depression, anxiety, bipolar disorder, schizophrenia, post-traumatic stress disorder, obsessive compulsive disorder, borderline personality disorder, and history of suicidal ideation

^10^Includes chronic liver conditions (such as cirrhosis, fatty liver disease, and chronic hepatitis, including viral hepatitis)

### Combinations of Underlying Conditions

Compared with having neither condition, having both heart disease and diabetes, obesity and diabetes, or hypertension and diabetes was significantly associated with an approximately threefold increased odds of death (Table 4). Patients with both heart disease and diabetes had 3.13 times (95% CI: 2.11, 4.62) the odds of death compared with those with neither heart disease nor diabetes. Patients with heart disease only had 1.97 times (95% CI: 1.30, 2.97) the odds of death and patients with diabetes only had 2.57 times (95 % CI: 1.33, 4.98) the odds of death compared with patients with neither condition. This pattern—patients with both conditions having increased odds of death compared with those having one or neither—was observed for all combinations considered.

**Table 4:** Matched adjusted odds ratios (aOR) of death with COVID-19 for study patients (N = 1029) with combinations of underlying medical conditions—New York City, March 13–April 9, 2020

Condition combinations^1^	Cases (n = 343)	Controls (n = 686)	Matched aOR^2^ (95% CI)
Heart disease^3^ and diabetes—n (%)			
Both	168 (49.0)	218 (31.8)	**3.13 (2.11, 4.62)**
Heart disease only	91 (26.5)	198 (28.9)	**1.97 (1.30, 2.97)**
Diabetes only	24 (7.0)	32 (4.7)	**2.57 (1.33, 4.98)**
Neither	60 (17.5)	238 (34.7)	Reference
Hypertension and diabetes—n (%)			
Both	156 (45.5)	195 (28.4)	**2.98 (2.06, 4.30)**
Hypertension only	76 (22.2)	156 (22.7)	**1.88 (1.25, 2.81)**
Diabetes only	36 (10.5)	55 (8.0)	**2.13 (1.25, 3.63)**
Neither	75 (21.9)	280 (40.8)	Reference
Hypertension and obesity—n (%)			
Both	151 (44.0)	200 (29.2)	**2.90 (1.94, 4.32)**
Hypertension only	81 (23.6)	151 (22.0)	**2.06 (1.35, 3.15)**
Obesity only	58 (16.9)	146 (21.3)	1.57 (0.99, 2.48)
Neither	53 (15.5)	189 (27.6)	Reference
Obesity and diabetes—n (%)			
Both	124 (36.2)	150 (21.9)	**2.91 (1.98, 4.28)**
Obesity only	85 (24.8)	196 (28.6)	**1.68 (1.13, 2.49)**
Diabetes only	68 (19.8)	100 (14.6)	**2.32 (1.49, 3.63)**
Neither	66 (19.2)	240 (35.0)	Reference
Hypertension and kidney disease^4^—n (%)			
Both	74 (21.6)	64 (9.3)	**3.81 (2.43, 5.98)**
Hypertension only	158 (46.1)	287 (41.8)	**1.81 (1.31, 2.51)**
Kidney disease only	5 (1.5)	7 (1.0)	2.10 (0.54, 8.18)
Neither	106 (30.9)	328 (47.8)	Reference
Obesity and kidney disease^4^—n (%)			
Both	49 (14.3)	40 (5.8)	**3.57 (2.17, 5.88)**
Obesity only	160 (46.6)	306 (44.6)	**1.63 (1.19, 2.24)**
Kidney disease only	30 (8.7)	31 (4.5)	**2.84 (1.56, 5.16)**
Neither	104 (30.3)	309 (45.0)	Reference

^1^Conditions listed in descending order of prevalence among case-patients

^2^Adjusted for sex, marital status, and insurance status. Bold-faced aORs indicate significance at alpha=0.05

^3^Includes history of heart attack, congestive heart failure, coronary artery disease, hypertension, hyperlipidemia, congenital heart disease, valvular heart disease, and other chronic heart conditions (such as angina, arrhythmias, cardiomyopathy, and pulmonary hypertension)

^4^Includes chronic kidney conditions (such chronic kidney disease, glomerulonephritis, and polycystic kidney disease)

### Race/Ethnicity and Age Stratification

The eight selected underlying conditions included in our stratified analysis had aOR point estimates >1 for death among all race/ethnicities, except lung disease among White patients (aOR, 0.85 [95% CI: 0.36, 1.98]) (Table 5). Five of these conditions were most prevalent among Black control-patients, including heart disease (74.9%), obesity (61.5%), diabetes (43.8%), lung disease (24.6%), and kidney disease (13.4%), compared with control-patients of other races/ethnicities. Magnitudes of association for conditions in groups aged <50 and ≥50 years were similar (Appendix 2).

**Table 5:** Matched adjusted odds ratios (aOR) of death with COVID-19 among study patients (N = 1029) with specific underlying medical conditions, stratified by race/ethnicity—New York City, March 13–April 9, 2020

Condition^1^	Cases (n = 343)	Controls (n = 686)	Matched aOR (95% CI)^2^
Any underlying condition—n (%)			
Non-Hispanic Asian			
Indicated	28 (90.3)	49 (81.7)	2.26 (0.50, 10.18)
Not indicated	3 (9.7)	11 (18.3)	Reference
Non-Hispanic Black			
Indicated	108 (99.1)	179 (95.7)	4.03 (0.46, 35.44)
Not indicated	1 (0.9)	8 (4.3)	Reference
Hispanic/Latino			
Indicated	114 (95.0)	214 (81.7)	**4.47 (1.84, 10.91)**
Not indicated	6 (5.0)	48 (18.3)	Reference
Non-Hispanic White			
Indicated	48 (100.0)	85 (86.7)	--^3^
Not indicated	0	13 (13.3)	Reference
Heart disease^4^—n (%)			
Non-Hispanic Asian			
Indicated	25 (80.6)	35 (58.3)	2.50 (0.84, 7.43)
Not indicated	6 (19.4)	25 (41.7)	Reference
Non-Hispanic Black			
Indicated	93 (85.3)	140 (74.9)	**2.36 (1.13, 4.94)**
Not indicated	16 (14.7)	47 (25.1)	Reference
Hispanic/Latino			
Indicated	79 (65.8)	133 (50.8)	**2.15 (1.31, 3.53)**
Not indicated	41 (34.2)	129 (49.2)	Reference
Non-Hispanic White			
Indicated	35 (72.9)	60 (61.2)	1.52 (0.65, 3.57)
Not indicated	13 (27.1)	38 (38.8)	Reference
Obesity—n (%)			
Non-Hispanic Asian			
Indicated	11 (35.5)	13 (21.7)	2.09 (0.71, 6.10)
Not indicated	20 (64.5)	47 (78.3)	Reference
Non-Hispanic Black			
Indicated	78 (71.6)	115 (61.5)	1.67 (0.93, 2.97)
Not indicated	31 (28.4)	72 (38.5)	Reference
Hispanic/Latino			
Indicated	73 (60.8)	133 (50.8)	**1.73 (1.10, 2.73)**
Not indicated	47 (39.2)	129 (49.2)	Reference
Non-Hispanic White			
Indicated	30 (62.5)	51 (52.0)	1.55 (0.74, 3.25)
Not indicated	18 (37.5)	47 (48.0)	Reference
Diabetes—n (%)			
Non-Hispanic Asian			
Indicated	16 (51.6)	24 (40.0)	1.42 (0.54, 3.72)
Not indicated	15 (48.4)	36 (60.0)	Reference
Non-Hispanic Black			
Indicated	72 (66.1)	82 (43.8)	**2.66 (1.55, 4.57)**
Not indicated	37 (33.9)	105 (56.2)	Reference
Hispanic/Latino			
Indicated	60 (50.0)	83 (31.7)	**1.97 (1.24, 3.12)**
Not indicated	60 (50.0)	179 (68.3)	Reference
Non-Hispanic White			
Indicated	24 (50.0)	31 (21.6)	2.07 (0.96, 4.49)
Not indicated	24 (50.0)	67 (68.6)	Reference
Lung disease^5^—n (%)			
Non-Hispanic Asian			
Indicated	5 (16.1)	6 (10.0)	2.28 (0.58, 8.98)
Not indicated	26 (83.9)	54 (90.0)	Reference
Non-Hispanic Black			
Indicated	32 (29.4)	46 (24.6)	1.14 (0.63, 2.09)
Not indicated	77 (70.6)	141 (75.4)	Reference
Hispanic/Latino			
Indicated	30 (25.0)	44 (16.8)	**1.97 (1.10, 3.54)**
Not indicated	90 (75.0)	218 (83.2)	Reference
Non-Hispanic White			
Indicated	12 (25.0)	24 (24.5)	0.85 (0.36, 1.98)
Not indicated	36 (75.0)	74 (75.5)	Reference
Kidney disease^6^—n (%)			
Non-Hispanic Asian			
Indicated	7 (22.6)	6 (10.0)	1.88 (0.51, 7.00)
Not indicated	24 (87.4)	54 (90.0)	Reference
Non-Hispanic Black			
Indicated	29 (26.6)	25 (13.4)	**2.59 (1.37, 4.90)**
Not indicated	90 (73.4)	162 (86.6)	Reference
Hispanic/Latino			
Indicated	30 (25.0)	26 (9.9)	**3.31 (1.75, 6.27)**
Not indicated	90 (75.0)	236 (90.1)	Reference
Non-Hispanic White			
Indicated	9 (18.8)	8 (8.2)	2.08 (0.66, 6.50)
Not indicated	39 (81.2)	90 (91.8)	Reference
Neurologic/neurodevelopmental condition^7^—n (%)			
Non-Hispanic Asian			
Indicated	2 (6.5)	4 (6.7)	1.20 (0.19, 7.60)
Not indicated	29 (93.5)	56 (93.3)	Reference
Non-Hispanic Black			
Indicated	24 (22.0)	19 (10.2)	**2.31 (1.09, 4.90)**
Not indicated	85 (78.0)	168 (89.8)	Reference
Hispanic/Latino			
Indicated	13 (10.8)	17 (6.5)	1.70 (0.75, 3.84)
Not indicated	107 (89.2)	245 (93.5)	Reference
Non-Hispanic White			
Indicated	19 (39.6)	11 (11.2)	**3.94 (1.57, 9.86)**
Not indicated	29 (60.4)	87 (88.8)	Reference
Mental health condition^8^—n (%)			
Non-Hispanic Asian			
Indicated	2 (6.5)	2 (3.3)	2.37 (0.30, 18.53)
Not indicated	29 (93.5)	58 (96.7)	Reference
Non-Hispanic Black			
Indicated	17 (15.6)	14 (7.5)	**2.42 (1.04, 5.61)**
Not indicated	92 (84.4)	173 (92.5)	Reference
Hispanic/Latino			
Indicated	9 (7.5)	14 (5.3)	1.57 (0.64, 3.87)
Not indicated	111 (92.50)	248 (94.7)	Reference
Non-Hispanic White			
Indicated	12 (25.0)	18 (18.4)	1.15 (0.47, 2.77)
Not indicated	36 (75.0)	80 (8.6)	Reference
Cancer—n (%)			
Non-Hispanic Asian			
Indicated	4 (12.9)	4 (6.7)	2.36 (0.52, 10.66)
Not indicated	27 (87.1)	56 (93.3)	Reference
Non-Hispanic Black			
Indicated	9 (8.3)	10 (5.3)	1.40 (0.54, 3.65)
Not indicated	100 (91.7)	177 (94.7)	Reference
Hispanic/Latino			
Indicated	12 (10.0)	11 (4.2)	**3.32 (1.35, 8.14)**
Not indicated	108 (90.0)	251 (95.8)	Reference
Non-Hispanic White			
Indicated	7 (14.6)	3 (3.1)	**4.43 (1.04, 18.79)**
Not indicated	41 (85.4)	95 (96.9)	Reference

^1^Any condition having a statistically significant aOR with approximately 10% prevalence among case-patients and any not statistically significant aOR of at least 1.5 and at least 15% prevalence among case-patients in unstratified analyses. Because of sparse data when stratifying, specific conditions that were part of grouped categories (e.g., heart disease, lung disease) were not presented on their own. Conditions listed in descending order by overall prevalence among case-patients

^2^Adjusted for patient sex, marital status, and insurance status. Bold-faced aORs indicate significance at alpha=0.05

^3^Undefined effect estimate

^4^Includes history of heart attack, congestive heart failure, coronary artery disease, hypertension, hyperlipidemia, congenital heart disease, valvular heart disease, and other chronic heart conditions (such as angina, arrhythmias, cardiomyopathy, and pulmonary hypertension)

^5^Includes asthma, chronic obstructive pulmonary disease, chronic bronchitis, emphysema, cystic fibrosis, and other chronic lung conditions (such as pulmonary nodules, pulmonary fibrosis, and history of pulmonary emboli)

^6^Includes chronic kidney conditions (such chronic kidney disease, glomerulonephritis, and polycystic kidney disease)

^7^Includes conditions such as seizure disorders, intellectual disability, dementia, history of traumatic brain injury, cerebral palsy, and neuropathy

^8^Includes conditions, such as attention deficit hyperactivity disorder, depression, anxiety, bipolar disorder, schizophrenia, post-traumatic stress disorder, obsessive compulsive disorder, borderline personality disorder, and history of suicidal ideation

## Discussion

This case-control study assessed risk factors for death among approximately 1000 persons aged 21–64 years hospitalized with COVID-19 across 82 NY metropolitan area hospitals. We found having any of the underlying conditions examined was associated with an over 4-fold increase in odds of death.

Black and Hispanic populations in NYC are at higher risk of COVID-19 compared with other racial and ethnic groups [13, 18, 19, 24]. Additionally, COVID-19 mortality rates in NYC are highest among Black and Hispanic persons [20]. In our study, 67% of case-patients were Black or Hispanic. Despite increased diagnosis and mortality rates, risk factors associated with greater in-hospital mortality among Black and Hispanic patients with COVID-19 have not been reported [13, 18, 19, 24]. We could not fully assess the association between race/ethnicity or poverty and in-hospital mortality because matching by neighborhood likely contributed to case-patients and control-patients having similar demographic and neighborhood-level characteristics. Race/ethnicity was not independently associated with mortality among study patients, yet racial/ethnic disparities in deaths were observed in NYC. Notably, the prevalence of five conditions was highest among Black control-patients. A similar pattern has been observed among NYC residents aged ≥18 years; the prevalence of hypertension, obesity, diabetes, and asthma among Black and Latino persons is higher compared with White and Asian persons [40]. Increased prevalence of conditions demonstrated to be associated with death likely contributed to the greater COVID-related mortality burden among Black and Latino persons in NYC.

In the U.S., White persons have a lower prevalence of diabetes and chronic kidney disease and lower rates of associated mortality compared with Black and Hispanic persons [41–43]. Racial/ethnic disparities associated with underlying condition prevalence and severe health outcomes can be explained by disparities in access to resources and opportunities, including health care [44, 45], housing [46], food [47], employment [44, 45], and education [48]. Further studies are needed to assess the associations between racial/ethnic disparities in these resources, SARS-CoV-2 infection, and severe outcomes. Additionally, research is needed to examine the associations between severe outcomes and (1) mitigation and severity of underlying conditions and (2) time to care.

Our findings may help guide the public health and medical communities’ efforts to prevent COVID-19 using health communication campaigns encouraging vaccine uptake, social distancing, face coverings, and handwashing. While citywide COVID-19 cases, hospitalizations, and deaths decline, and vaccination rates rise, racial/ethnic disparities persist. As of June 2021, only 30% of Black and 39% of Hispanic NYC adult residents were fully vaccinated, compared with 47% of White and 67% of Asian NYC adult residents [49]. These observed racial/ethnic disparities, including those in underlying condition prevalence, underscore the importance of prioritizing and increasing COVID-19 prevention efforts in these communities. Such efforts may include provision of additional social services, increased health resources, and especially vaccination efforts for racial and ethnic minority groups, given the increased prevalence of underlying conditions that are risk factors for death with COVID-19 and the increased burden of overall mortality experienced by Black and Hispanic populations both during and after our study period [1].

Our study has limitations. First, the multiple EHR systems did not consistently capture information. Although known to be associated both with certain underlying conditions [50, 51] and death [52, 53], substance use and occupation could not be analyzed given incomplete data, which is a known limitation to EHR data [54]. Additionally, BMI was missing from 22% of study patients, potentially limiting our ability to distinguish risk among BMI categories. Second, clinicians may have had fewer opportunities to ascertain complete medical histories for case-patients before their deaths. By combining patients who did not have certain conditions with those for whom information was not captured, our observed effect sizes are likely more conservative than the true associations. Third, while control-patients were discharged and not known to have died as of July 1, 2020, some might have been discharged to locations providing a higher level of care than they received prior to hospital admission for COVID-19 and/or might have experienced persistent or new symptoms related to COVID-19; such patients would be less likely to represent healthy controls, and observed associations between underlying conditions and death might be more conservative than true estimates. Fourth, stratified analyses by race/ethnicity were underpowered, and lack of observed significant associations should not be interpreted as no association. Fifth, we did not account for severity or control of underlying conditions, nor did we collect dosing information about inpatient treatments, so we cannot assess differences in association and death by these factors. Finally, generalizability of our results might be limited; our study was restricted to one city and hospitalized patients, and conducted early in the pandemic when the DOHMH had temporary surge staffing resources and remote EHR access, and when clinical management and treatment guidance was rapidly evolving. Despite resource constraints, which precluded us from extending the study period, findings nonetheless help explain why COVID-related health inequities persist within NYC.

Following the study period, COVID-19 mortality rates declined in NYC [20]. COVID-19 treatment guidelines [55] have evolved since the study period, which likely contributed to improved clinical decision-making and inpatient management. These circumstances reinforced our decision to only describe, and not conduct statistical tests on, the clinical characteristics of our study patients. Additionally, public health messaging in NYC shifted since the beginning of the pandemic, encouraging patients with severe symptoms to promptly seek care [56]. Obtaining comprehensive medical histories for hospitalized patients aged 21–64 years with COVID-19 is critical and may inform clinical decision-making since underlying conditions, particularly combinations of conditions, are risk factors for in-hospital mortality. Finally, observed racial/ethnic disparities in underlying condition prevalence, COVID-19 diagnosis, hospitalization, and mortality suggest the importance of increasing COVID-19 prevention efforts, especially among racial/ethnic minorities.

## Data Availability

Line-level patient data are not publicly available in accordance with patient confidentiality and privacy laws. Public data are available from: https://www1.nyc.gov/site/doh/covid/covid-19-data.page This analysis used standard conditional logistic regression techniques and did not require custom code. Sample code is provided in Appendix 3.

## Appendix 1. Sample size calculation details

Based on prevalence estimates of two underlying conditions of interest, diabetes and obesity, in the general NYC adult population [56] and among patients diagnosed with COVID-19 in NYC- and US-based studies [17–21], the expected prevalence of diabetes and of obesity for control-patients was 18% and 42%, respectively. A sample size of 293 case-patients (for diabetes, yielding the larger minimum number of the two conditions) and two matched control-patients per case-patient, assuming the correlation coefficient for having diabetes between matched case- and control-patients was 0.20, would achieve 80% power to detect a moderate effect size (i.e., odds ratio for diabetes prevalence of 1.70 versus the alternative of equal odds) using a chi-square test with α = 0.05. Thus, we aimed to include at least 300 case-patients and 600 matched control-patients.

### Appendix 2. Matched adjusted odds ratios (aOR) of death with COVID-19 among study patients (N = 1029) with specific underlying medical conditions, stratified by age group—New York City, March 13–April 9, 2020

**Table:**

Condition^1^	Cases (n = 343)	Controls (n = 686)	Matched aOR (95% CI)^2^
Any underlying condition—n (%)			
< 50 years			
Indicated	84 (93.3)	140 (77.8)	**3.71 (1.46, 9.42)**
Not indicated	6 (6.7)	40 (22.2)	Reference
≥ 50 years			
Indicated	247 (97.6)	53 (10.5)	**5.05 (2.04, 12.50)**
Not indicated	6 (2.37)	453 (89.5)	Reference
Any heart disease^3^—n (%)			
< 50 years			
Indicated	51 (56.7)	62 (34.4)	**2.26 (1.29, 3.95)**
Not indicated	39 (43.3)	118 (65.6)	Reference
≥ 50 years			
Indicated	208 (82.2)	354 (70.0)	**2.07 (1.37, 3.13)**
Not indicated	45 (17.8)	152 (30.0)	Reference
Obesity—n (%)			
< 50 years			
Indicated	58 (64.4)	95 (52.8)	**1.95 (1.10, 3.47)**
Not indicated	32 (35.6)	85 (47.2)	Reference
≥ 50 years			
Indicated	151 (59.7)	251 (49.6)	**1.53 (1.11, 2.12)**
Not indicated	102 (40.3)	255 (50.4)	Reference
Diabetes—n (%)			
< 50 years			
Indicated	36 (40.0)	39 (21.7)	**2.29 (1.27, 4.13)**
Not indicated	54 (60.0)	141 (78.3)	Reference
≥ 50 years			
Indicated	156 (61.7)	211 (41.7)	**2.03 (1.46, 2.84)**
Not indicated	97 (38.3)	295 (58.3)	Reference
Any lung disease^4^—n (%)			
< 50 years			
Indicated	22 (24.4)	40 (22.2)	1.06 (0.54, 2.11)
Not indicated	68 (75.6)	140 (77.8)	Reference
≥ 50 years			
Indicated	68 (26.9)	91 (18.0)	**1.73 (1.16, 2.58)**
Not indicated	185 (73.1)	415 (82.0)	Reference
Any kidney disease^5^—n (%)			
< 50 years			
Indicated	14 (15.6)	9 (5.0)	**3.05 (1.18, 7.84)**
Not indicated	76 (84.4)	171 (95.0)	Reference
≥ 50 years			
Indicated	65 (25.7)	62 (12.3)	**2.38 (1.55, 3.58)**
Not indicated	188 (74.3)	444 (87.7)	Reference
Any neurologic/neurodevelopmental condition^6^—n (%)			
< 50 years			
Indicated	17 (18.9)	12 (6.7)	**3.55 (1.40, 8.98)**
Not indicated	73 (81.1)	168 (93.3)	Reference
≥ 50 years			
Indicated	48 (19.0)	44 (8.7)	**2.16 (1.33, 3.49)**
Not indicated	205 (81.0)	462 (91.3)	Reference
Any mental health diagnosis^7^—n (%)			
< 50 years			
Indicated	10 (11.1)	10 (5.6)	1.84 (0.69, 4.86)
Not indicated	80 (88.9)	170 (94.4)	Reference
≥ 50 years			
Indicated	33 (13.0)	43 (8.5)	1.55 (0.92, 2.61)
Not indicated	220 (87.0)	463 (91.5)	Reference
Cancer			
< 50 years			
Indicated	5 (5.6)	2 (1.1)	4.16 (0.74, 23.33)
Not indicated	85 (94.4)	178 (98.9)	Reference
≥ 50 years			
Indicated	32 (12.7)	30 (5.9)	**2.34 (1.36, 4.03)**
Not indicated	221 (87.3)	476 (94.1)	Reference

^1^Any condition having a statistically significant aOR with approximately 10% prevalence among case-patients and any not statistically significant aOR of at least 1.5 and at least 15% prevalence among case-patients in unstratified analyses. Because of sparse data when stratifying, specific conditions that were part of grouped categories (e.g., heart disease, lung disease) were not presented on their own. Conditions listed in descending order by overall prevalence among case-patients

^2^Adjusted for patient sex, marital status, and insurance status. Bold-faced aORs indicate significance at alpha=0.05

^3^Includes history of heart attack, congestive heart failure, coronary artery disease, hypertension, hyperlipidemia, congenital heart disease, valvular heart disease, and other chronic heart conditions (such as angina, arrhythmias, cardiomyopathy, and pulmonary hypertension)

^4^Includes asthma, chronic obstructive pulmonary disease, chronic bronchitis, emphysema, cystic fibrosis, and other chronic lung conditions (such as pulmonary nodules, pulmonary fibrosis, and history of pulmonary emboli)

^5^Includes chronic kidney conditions (such as chronic kidney disease, glomerulonephritis, and polycystic kidney disease)

^6^Includes conditions such as seizure disorders, intellectual disability, dementia, history of traumatic brain injury, cerebral palsy, and neuropathy

^7^Includes conditions, such as attention deficit hyperactivity disorder, depression, anxiety, bipolar disorder, schizophrenia, post-traumatic stress disorder, obsessive compulsive disorder, borderline personality disorder, and history of suicidal ideation

## Appendix 3. Sample code looking at interaction analyses by race/ethnicity

proc logistic data=analysis;

class pc_exists (ref="No") raceeth (ref="White") gender (ref="FEMALE")

ins (ref="Private") marital (ref="Married") / param=ref;

model outbreak_inv_status (event="Case") = pc_exists raceeth

pc_exists*raceeth gender ins marital;

strata strata;

oddsratio pc_exists / at(raceeth="Asian");

oddsratio pc_exists / at(raceeth="Black");

oddsratio pc_exists / at(raceeth="Hispanic");

oddsratio pc_exists / at(raceeth="White");

**run**;

/*variable names*/

*pc_exists: presence of any underlying condition;

*raceeth: patient race/ethnicity;

*gender: patient sex;

*ins: patient insurance status;

*marital: patient marital status;

*outbreak_inv_status: case vs. control status;

*strata: pooled strata identifier;
